# Tetrac Delayed the Onset of Ocular Melanoma in an Orthotopic Mouse Model

**DOI:** 10.3389/fendo.2018.00775

**Published:** 2019-01-08

**Authors:** Osnat Ashur-Fabian, Ofira Zloto, Ina Fabian, Galya Tsarfaty, Martin Ellis, David M. Steinberg, Aleck Hercbergs, Paul J. Davis, Ido Didi Fabian

**Affiliations:** ^1^Department of Human Molecular Genetics and Biochemistry, The Sackler Faculty of Medicine, Tel Aviv University, Tel Aviv, Israel; ^2^Translational Hemato-Oncology Laboratory, Meir Medical Center, The Hematology Institute and Blood Bank, Kfar-Saba, Israel; ^3^Goldschleger Eye Institute, Sheba Medical Center, Affiliated to The Sackler Faculty of Medicine, Tel Aviv University, Tel Aviv, Israel; ^4^Department of Cell and Developmental Biology, The Sackler Faculty of Medicine, Tel Aviv University, Tel Aviv, Israel; ^5^Department of Diagnostic Imaging, Sheba Medical Center, Ramat Gan, Israel; ^6^Sackler Faculty of Medicine, Tel Aviv University, Tel Aviv, Israel; ^7^Department of Statistics and Operations Research, Tel Aviv University, Tel Aviv, Israel; ^8^Department of Radiation Oncology, Cleveland Clinic, Cleveland, OH, United States; ^9^Pharmaceutical Research Institute, Albany College of Pharmacy and Health Sciences, Albany, NY, United States; ^10^Department of Medicine, Albany Medical College, Albany, NY, United States

**Keywords:** melanoma, mouse, αvβ3 integrin, thyroid, tetrac

## Abstract

Ocular melanoma research, the most common primary intraocular malignancy in adults, is hindered by limited *in vivo* models. In a series of experiments using melanoma cells injected intraocularly into mouse eyes, we developed a model for ocular melanoma. Inoculation of 5 × 10^5^ B16F10 cells led to rapid tumor growth, extensive lung metastasis, and limited animal survival, while injection of 10^2^ cells was sufficient for intraocular tumors to grow with extended survival. In order to improve tumor visualization, 10^2^ melanoma cells (B16F10 or B16LS9) were inoculated into Balb/C albino mouse eyes. These mice developed intraocular tumors that did not metastasize and exhibited extended survival. Next, we studied the therapeutic potential of inhibitor of the thyroid hormones-αvβ3 integrin signaling pathway in ocular melanoma. By utilizing tetraiodothyroacetic acid (tetrac), a thyroid hormone derivative, a delay in tumor onset in the B16F10 (integrin+) arm was observed, compared to the untreated group, while in the B16LS9 cells (integrin–) a similar rate of tumor onset was noticed in both experimental and control groups. In summary, following an optimization process, the mouse ocular melanoma model was developed. The models exhibited an extended therapeutic window and can be utilized as a platform for investigating various drugs and other treatment modalities.

## Introduction

Ocular melanoma is the most common primary intraocular malignancy in adult patients (1, 2). It is estimated that about 50% of patients develop metastatic spread, predominantly to the liver (3, 4). There are no effective treatments and death results in ~1 year following detection of systemic involvement (5). Although diagnostic and therapeutic tools for the primary ocular tumor have improved significantly over the past 40 years, there has been no change in survival rates (4, 6), emphasizing the need for alternatives to traditional treatments.

Animal models play a significant role in understanding tumor development as well as for developing novel therapeutic approaches in preclinical studies. Efforts have been made to generate ocular melanoma animal models that are suitable for uveal melanoma and its distinctive metastatic behavior (7). However, ocular melanoma research is still hindered by limited *in vivo* models and development is needed of a model that may provide a therapeutic window for preclinical evaluation of experimental treatments.

Thyroid hormones have been shown to influence tumor growth and angiogenesis in a variety of cancer models (8). These effects are attributed to the non-genomic hormonal effects [reviewed in Cheng et al. (9) and Davis et al. (10, 11)]. One of the mechanisms whereby such non-genomic actions may be mediated is via binding of the thyroid hormone to the extracellular domain of integrin αvβ3 (12), a protein which is overexpressed in an array of cancer types and correlates with disease stage (13). Upon binding, thyroid hormone, primarily L-thyroxine (T_4_), induces diverse membrane-initiated intracellular activities [reviewed in Davis et al. (11)], including cell proliferations, mainly via the MAPK pathway. Such mitogenic activities have been shown in various types of cancer cells, including glioma (14), breast cancer (15), hepatocarcinoma (16), thyroid cancer (17), sarcoma (18), tumor-associated vascular cells (19), myeloma (20–22) and ovarian cancer (23). We have recently established that hyperthyroidism shortened survival time in a metastatic ocular melanoma mouse model, while hypothyroidism had a significant protective effect (24). Based on these collective results, we hypothesized that natural thyroid hormone derivatives with low-potency thyromimetic activity at the integrin may be utilized for growth inhibition in ocular melanoma. Such analog includes a deaminated form of T_4_, tetraiodothyroacetic acid (tetrac), which possess low hormone activity because of shortening of the side chain on the inner ring (removal of a carbon or amine), resulting in the conversion of propionic acid (thyroid hormone) to acetic acid (tetrac). This transforms the compound from thyroid agonists to antagonist (10). Tetrac has low affinity for the nuclear thyroid hormone receptors, through which the classical genomic actions are initiated by the thyroid hormone and is a low-grade thyromimetic in the nucleus (9). Such low-grade thyromimetic genomic effects of tetrac have been shown in various tissues [e.g., (25–27)] and high rates of liver glucuronidation of triac and tetrac have been thought to explain their low bioactivity (26). In contrast, tetrac is an antagonist of T4 actions at the hormone receptor on the extracellular domain of integrin αvβ3 (11). At the cell surface integrin receptor tetrac was shown to displace thyroid hormones binding and to block αvβ3, resulting in reduced cell proliferation, anti-angiogenesis and reduced anti-apoptotic defense pathways activity in multiple cancer models, including mice and human melanoma (28, 29) and reviewed in Davis et al. (11). This antitumor activity of tetrac is initiated at the integrin and chemical modification of tetrac to prevent its nuclear uptake and thus restrict its action to the receptor on αvβ3 heightens the anticancer activity of tetrac via the membrane receptor.

We herein report the development of novel mouse models of ocular melanomas and the effect of a specific thyroid hormone-integrin antagonist on delaying the onset of tumor growth in such models.

## Materials and Methods

### Reagents

Tetrac (Sigma-Aldrich, St. Louis, MO, USA) was dissolved in 0.04 N KOH 4% propylene glycol (PG) solution to a concentration of 1 mg/1 mL.

### Cell Lines

B16F10 mouse melanoma cell line (ATCC, CRL-6475) and B16LS9 (a generous gift from Grossniklaus Hans E, MD, Emory Eye Center, Atlanta, GA, USA) were cultured in RPMI 1,640 medium, supplemented with 10% (v/v) heat inactivated fetal calf serum, 2 mM L-glutamine and antibiotics (penicillin/streptomycin), in a humidified atmosphere of 5% CO_2_ at 37°C.

### Expression of Integrin αvβ3

The expression of αvβ3 integrin on B16F10 or B16Ls9 melanoma cells was quantified using flow-cytometry. In details, the melanoma cells (100,000 cells) were harvested and labeled with 50 μg/mL PE-αv antibodies (Clone RMV-7, Abcam), and FITC-β3 antibodies (Clone HM beta 3.1, Abcam) in 100 mL phosphate-buffered saline (PBS). Following incubation for 15 min at room temperature, the cells were centrifuged, diluted in PBS, and analyzed by a flow cytometer (MACSQuant, Miltenyi Biotec, Bergisch Gladbach, Germany).

### Animals

Study animals were wild-type male C57BL/6 or Balb/C mice aged 8 weeks (Harlan Laboratories Ltd, Ein Kerem, Jerusalem). Mice were maintained under specific pathogen-free conditions and housed under controlled conditions (temperature: 20–24°C; humidity: 60–70%). The mice were acclimated to our vivarium for 1 week prior to their use according to study protocols. Up to 6 animals were housed in a cage under conventional conditions and fed chow and water *ad libitum*. All animal procedures and experiments were conducted with approval and under the supervision of the Institutional Animal Care Committee at Tel-Aviv University, and conformed to recommendations of the Association for Research in Vision and Ophthalmology Statement for the Use of Animals in Ophthalmic and Vision Research.

### Experimental Groups and Inoculation of Tumor Cells

For model optimization in the C57Bl/6 mice, the subretinal space (i.e., the choroid) of each mouse's right eye was first inoculated with aliquots of 5 × 10^5^ B16F10 cells (*n* = 8 mice). Next, the same cells were inoculated at decreasing concentration (10^4^, 10^3^, and 10^2^ cells, total 15 mice, *n* = 5 for each concentration). For assessing the Balb/C Albino mice model, the subretinal space of each mouse's right eye was inoculated with aliquots of 10^2^B16F10 or B16LS9 cells/1 μL PBS (*n* = 5 for each cell type), using a transconjunctival approach as previously described (30), allowing the inoculated cells to remain in the eye. Mice were anesthetized with a mixture of ketamine and xylazine (120 mg/kg ketamine, 10 mg/kg xylazine), and the experimental eye was desensitized by a drop of oxybuprocaine (Dr. Fischer, Bnei Barak, Israel). Under a dissecting microscope, a 30-gauge needle was inserted ~1 mm posterior to the limbus through the conjunctiva and sclera and into the subretinal space. The tip of a 10 μL glass syringe with a 32-gauge blunt needle (Hamilton Co., Bonaduz, Switzerland) was introduced into the subretinal space via the needle track, and a 1 μL suspension of tumor cells was then injected into the eyes of the animals. No cells were inoculated until the needle tip was inside the eye, no tumor cell reflux occurred, and the subconjunctival space remained free of tumor cells. For the final interventional study, the subretinal space of each Balb/C Albino mouse's right eye was inoculated with aliquots of 10^2^B16F10 or B16LS9 cells in 1 μL PBS. For each experimental model, mice were given drinking water with 35 μg tetrac per day (*n* = 16 mice in the B16F10 model and *n* = 16 in the B16LS9 model), whereas the control group mice were given only polyethylene glycol dissolved in water (*n* = 15 mice in the B16F10 model and *n* = 13 mice in the B16LS9 model). Drinking water was exchanged on daily basis.

### Clinical Follow-Up

Mice were checked daily for clinical evidence of intraocular tumor growth. These signs appeared in the form of intraocular bleeding, turbidity, or both. When any of these signs became evident, the mouse was transferred to a separate cage and followed-up until death. The interval between inoculation of tumor cells and death was defined as the survival time. The interval between inoculation of tumor cells and first clinical evidence of intraocular tumor growth was defined as the inoculation-to-tumor time, and the interval between first clinical evidence of intraocular tumor growth to death was defined as the tumor-to-death time. All of these data were recorded and analyzed. We did not observe an effect of the treatments on animal bodyweight (data not shown), an index of lack of toxicity.

### Ultrasound and Doppler Measurements

Following general and local anesthesia, as mentioned above, Aquasonic Clear Ultrasound Gel (Medthechnica Healthcare Solutions, Petah-Tikva, Israel) was applied on the mouse's eye baring the intraocular tumor and tumor dimensions were measured and blood flow recorded using an ultrasound probe [Sequoia 512 (Acuson, Mountain View, California, US)] or Vevo 2100 (VisualSonics, Toronto, Canada). Imaging results were analyzed thereafter.

### Computed Tomography Scan

Following general anesthesia, as mentioned above, and after injection of Omnipaque™ (iohexol, GE Healthcare, Tel Aviv, Israel) as contrast into the mouse tail vein, mice underwent a CT scan using a TomoScope® Synergy deivce (CT-Imaging, Erlangen, Germany) focusing on the lungs. Serial images were analyzed for the presence of metastases.

### Histopathological and Immunohistochemical Studies

The tumor-bearing eyes of all the inoculated mice and lungs of mice from each experimental group were harvested and sent for pathological and immunohistochemical evaluations. Formalin-fixed, paraffin-embedded sections of the collected specimens were hematoxylin and eosin (H&E) stained for histopathologic assessment. For immunostaining, the slides were warmed to 60°C for 60 min, dewaxed in xylene and rehydrated. Hydrogen peroxide (H_2_O_2_, 3% in PBS) was used to block endogenous peroxidase activity. After being rinsed in PBS, the sections were incubated for 60 min at room temperature with anti-S100 (Z0311, 1:1,000, Dako, Herzliya, Israel), a melanoma marker or anti β3 integrin antibody (ab75872, Abcam, Cambriodge, UK). Detection was performed with Envision+ System-HRP Labeled Polymer Anti-Rabbit (K4003, Dako). The binding antibody was visualized with chromogen AEC substrate (Invitrogen Corporation). Sections were counterstained with hematoxylin and cover-slipped with an aqueous mounting fluid (Glycerol, Dako). The stained sections were reviewed with a light microscope and analyzed by a pathologist.

### Statistics

Analysis of the delay in tumor onset was done using the non-parametric logrank test (Mantel-Cox test). Results were considered statistically significant for a *p* < 0.05.

## Results

### Optimization of Orthotopic Mouse Ocular Melanoma Models

**Figure 1:**
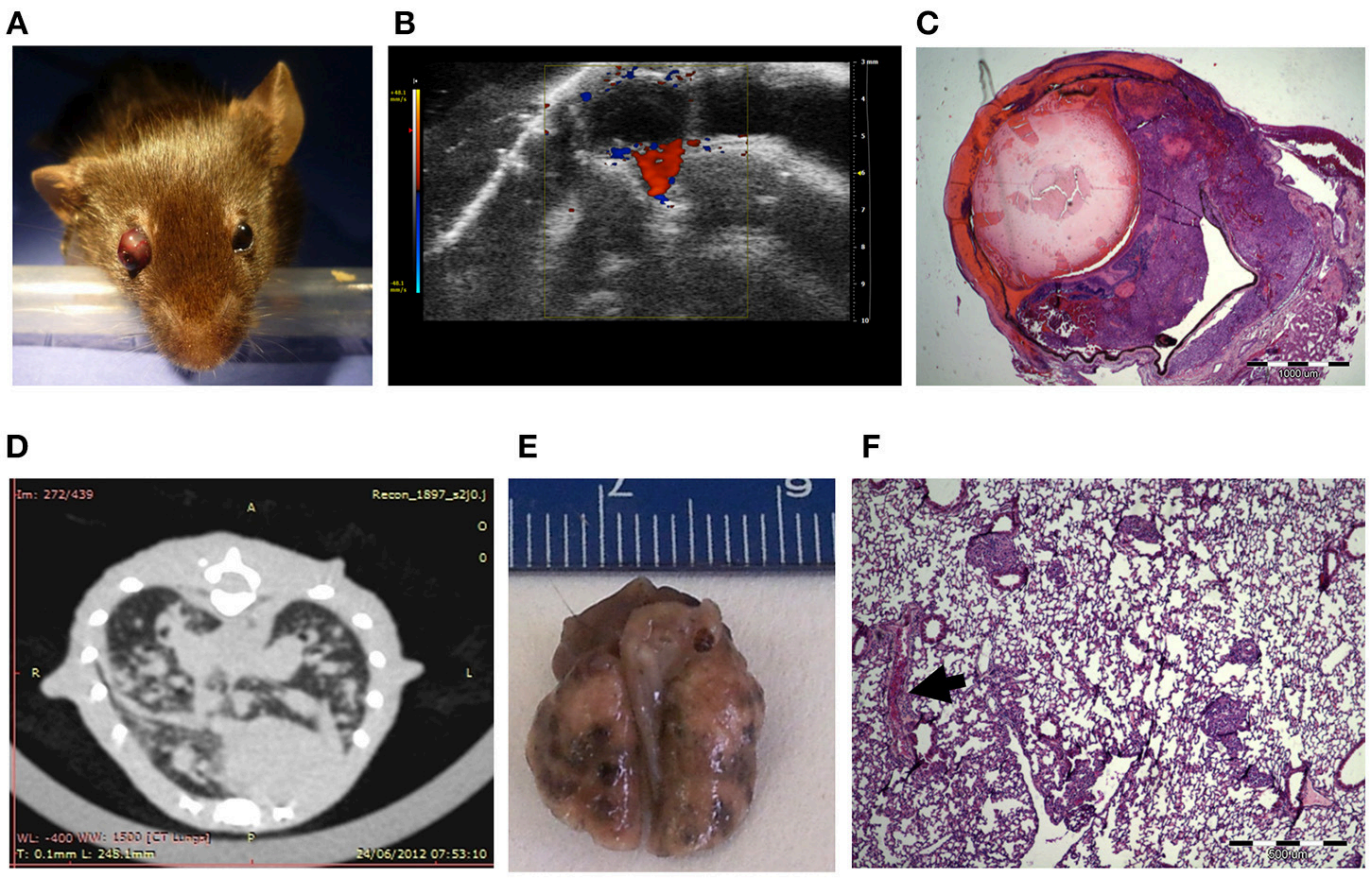
C57Bl/6 mice inoculated with 5 × 10^5^ B16F10 cells **(A)** Buphthalmic (enlarged) right eye filled with tumor. **(B)** Ultrasound Doppler demonstrating blood flow in an intraocular melanoma. **(C)** H&EX2 staining from an enucleated eye. **(D)** CT scan showing lung metastasis. **(E)** Macro metastasis of B16F10 cells in the lung of a representative mouse. **(F)** An aggregate of large epithelioid melanoma cells with expanded cytoplasm, large nuclei and prominent nucleoli within it (arrow), is surrounded by typical lung tissue (H&EX40).

For the generation of an ocular melanoma mouse model, we used the mouse melanoma B16F10 cell line, given its ability to form intraocular tumors (31–34). By injecting 5 × 10^5^ B16F10 cells into the posterior segment of C57Bl/6 murine eyes (*n* = 8), a tumor occupying the entire intraocular cavity was observed within 5–7 days from inoculation (Figure 1A). In addition, ultrasound Doppler evaluation showed blood flow within the mass (Figure 1B).

After 10 days, eyes were enucleated and sent for pathological processing and H&E staining. Figure 1C depicts characteristic melanoma cells behind the lens, between the pigment epithelium and retina in a representative eye specimen.

As the B16F10 cell line is known to metastasize predominantly to the lungs (35), animal CT scans were performed, clearly showing lung metastasis (Figure 1D). Macro-metastasis of B16F10 cells, surrounded by typical lung tissue, is shown in dissected lungs from a representative mouse (Figure 1E). Pathological processing and H&E staining of the dissected lungs demonstrated aggregates of large epithelioid melanoma cells, surrounded by typical lung tissue (Figure 1F). This mouse model in summary showed that intraocular tumor cell growth was rapid and resulted in extensive lung metastasis and limited survival within about 2 weeks. These findings restricted the applicability of this model tor the evaluation of anti-cancer treatments.

**Figure 2:**
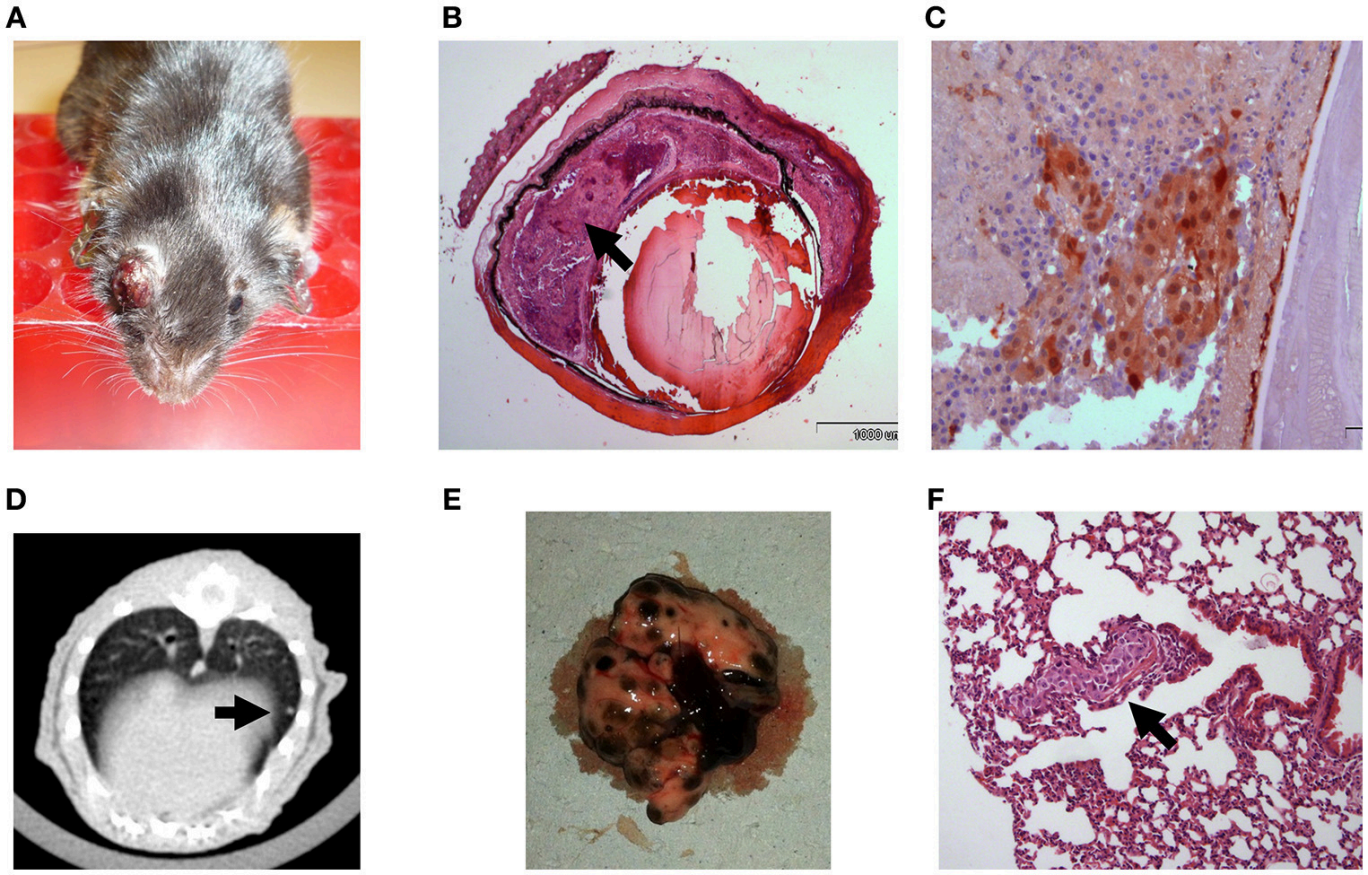
C57Bl/6 mice inoculated with 100 B16F10 cells **(A)** Buphthalmic (enlarged) right eye filled with tumor. **(B)** Enucleated murine eye showing the intraocular tumor located behind the lens, between the pigment epithelium and retina (H&EX2, arrow). **(C)** Tumor cells behind the lens labeled for S100(X20). **(D)** CT scan with lung metastasis **(E)** Macro metastasis in the lungs. **(F)** An aggregate of large epithelioid melanoma cells with expanded cytoplasm, large nuclei and prominent nucleoli within it (arrow), is surrounded by typical lung tissue (H&EX40).

In order to establish a mouse model in which tumor growth rate and metastasis permit a longer therapeutic window, we gradually reduced the intraocular-injected melanoma cell amounts. Aliquots of B16F10 cells were scaled-down to 10^4^, 10^3^, and eventually 10^2^ cell aliquots (*n* = 5 mice in each group) and injected into C57Bl/6 mouse eyes. Based on this preliminary optimization process, the 10^2^ cell aliquot was chosen for further use in our experiments because the tumors developed relatively slowly, with a wider therapeutic window. Results indicated that an injection of 10^2^ B16F10 cells is sufficient to cause development of a tumor (Figure 2A) between 14 and 17 days post inoculation. The enucleated eyes exhibited an intraocular tumor behind the lens (Figure 2B). Positive S100 immunostaining confirmed the presence of melanoma cells (Figure 2C). Similar to the results with inoculation of 5 × 10^5^ B16F10 cells, in this model, macro metastasis in the lungs were documented by CT (Figure 2D) in the dissected lungs (Figure 2E), and by H&E staining (Figure 2F). Taken together, the inoculation of 10^2^ melanoma cells resulted in a mouse model which exhibited slower rate of intraocular tumor growth and metastasis and thus extended the therapeutic window required for pre-clinical anticancer drug evaluations. We have successfully used this model for studying the role of thyroid hormones on ocular melanoma growth (24).

### Albino Mouse Ocular Melanoma Model Exhibit Extended Survival and No Tumor Metastasis

**Figure 3:**
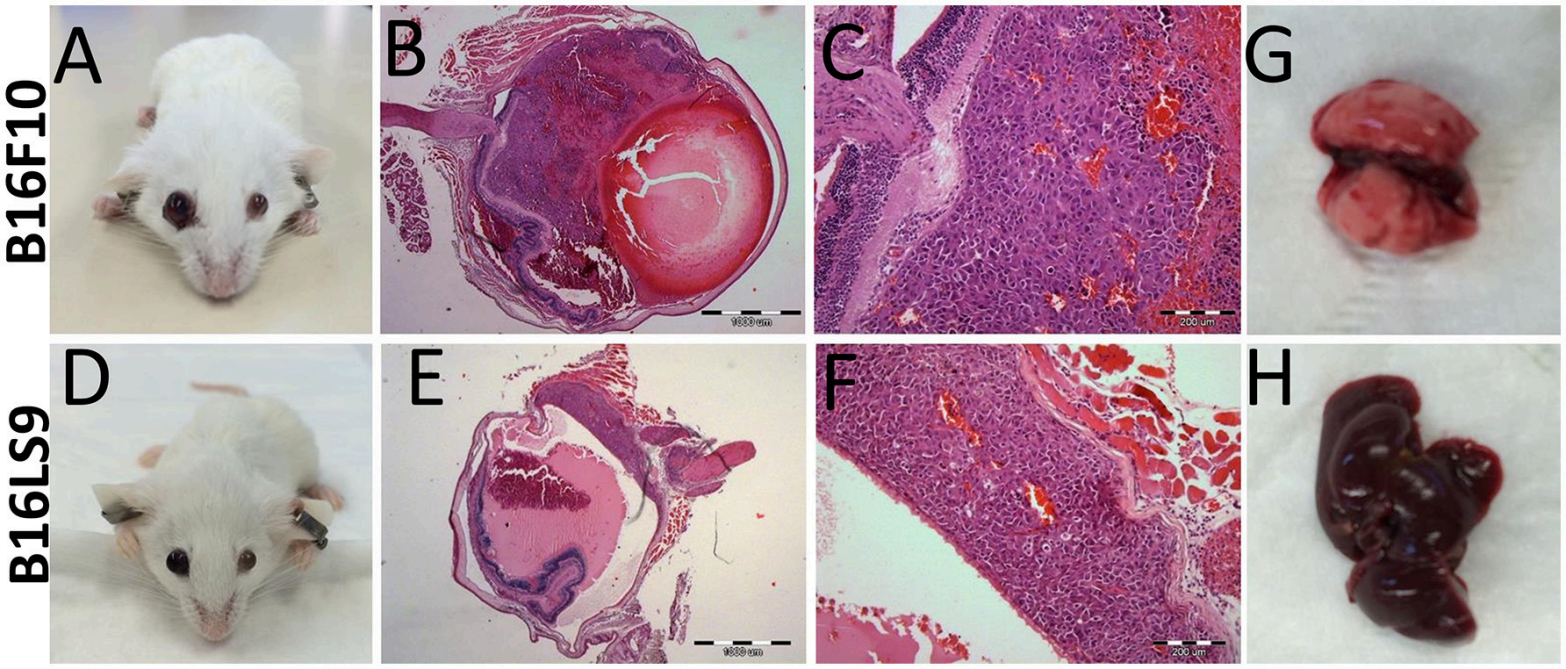
BALB/c White mice inoculated with **(A–C)** 100 B16F10 cells and **(D–F)** 100 B16LS9 cells. **(A,D)** Buphthalmic (enlarged) right eye filled with tumor. **(B,E)** Enucleated murine eye showing the intraocular tumor located behind the lens (H&EX2). **(C,F)** H&EX10 **(G)** Representative lungs from the B16F10 model **(H)** Representative liver from the B16LS9 model.

One of the limitations of using C57Bl/6 mice as an animal model in this context was its dark eyes, which make it difficult to detect the pigmented intraocular tumors at early stages. We therefore used, as a next step, another mouse strain, the Balb/C albino, which we anticipated would allow better visualization of the intraocular tumors. We repeated the same protocol of inoculating 10^2^ melanoma cells into the albino mouse eyes. In the albino mouse studies, an additional mouse melanoma cell line was utilized: B16LS9 (*n* = 5 for each model). While B16F10 cells metastasize primarily to the lungs, the B16LS9 cells are known to spread to the liver when implanted into mice eyes (7, 36). Enlarged eye filled with tumor resulted; starting from 2 weeks following intraocular injections of 10^2^ B16F10 cells (Figure 3A) or B16LS9 cells (Figure 3D). Similarly, the enucleated eyes from both models exhibited intraocular tumor behind the lens, between the pigment epithelium and the retina (Figures 3B,C,E,F). Interestingly, inoculation of 10^2^ B16F10 or B16LS9 cells in the albino mice did not result in tumor metastasis in the lungs (Figure 3G) or liver (Figure 3H). These mice continued to thrive for up to 3 months, after which the experiment was terminated, according to our animal ethics protocol.

### Tetrac Delayed the Onset of Ocular Melanoma in the Albino Mouse Model

The B16F10 cells serve as a valid platform to examine the thyroid-hormone-αvβ3 axis *in vivo*, due to high expression of this specific integrin (Supplementary Figures S1A–D). In contrast, the B16LS9 cells express low levels of αvβ3 (Supplementary Figures S1E–H). We have recently established in a B16F10 ocular melanoma model (24) that the hypothyroid environment enhances survival of mice inoculated with the B16F10 cells, while hyperthyroidism resulted with shorter survival. The unexpected observation that albino mice, when inoculated intraocularly with melanoma cells, do not develop metastasis and exhibit an extended survival, led us to exploit these models to study the potential of thyroid-hormone-αvβ3 inhibitors in delaying the onset of ocular melanoma. One such inhibitor that has been shown in numerous *in vitro* and *in vivo* studies to inhibit thyroid hormone binding to αvβ3 integrin is tetrac and this agent was selected for the next study.

**Figure 4:**
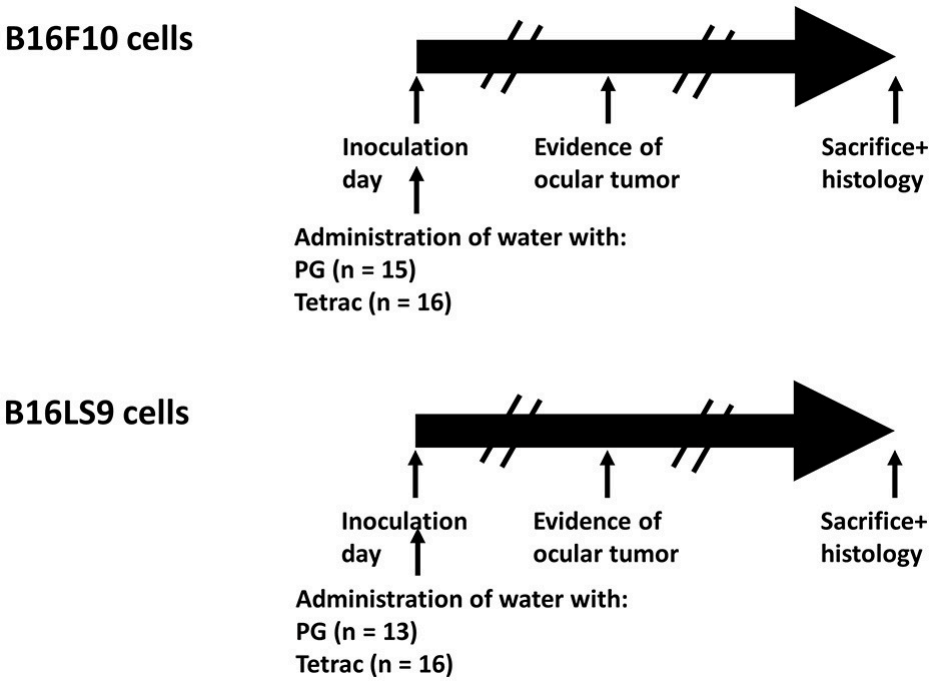
Experimental design. BALB/c mice were inoculated into the right eye subretinal space with aliquots of B16F10 or B16LS9 cells (10^2^ cells/1 μL PBS). Each experimental tumor model was divided into mice given tap water with propylene glycol (Control group) or to mice given drinking water containing tetrac (Experimental group). The number of mice in each group is indicated. The mice were monitored on a daily basis and first sign of intraocular tumor growth was recorded. The mice were followed-up and recorded for tumor initiation for 37–40 days.

The subretinal space of the right eye of BALB/c mice was inoculated with aliquots of 10^2^ B16F10 or B16LS9 cells/1 μL PBS (inoculation day or day 0) using a transconjunctival approach, as previously described (30). There were no cases of cell reflux following tumor inoculation and the subconjunctival space remained free of tumor cells. On the same day, each experimental tumor model was divided into mice given tap water (Control group) or drinking water containing tetrac. The mice were monitored on a daily basis and the first sign of intraocular tumor growth was recorded. The experiment design, including the number of mice in each group is indicated in Figure 4. The mice were followed-up and recorded for tumor initiation for 37–40 days.

**Figure 5:**
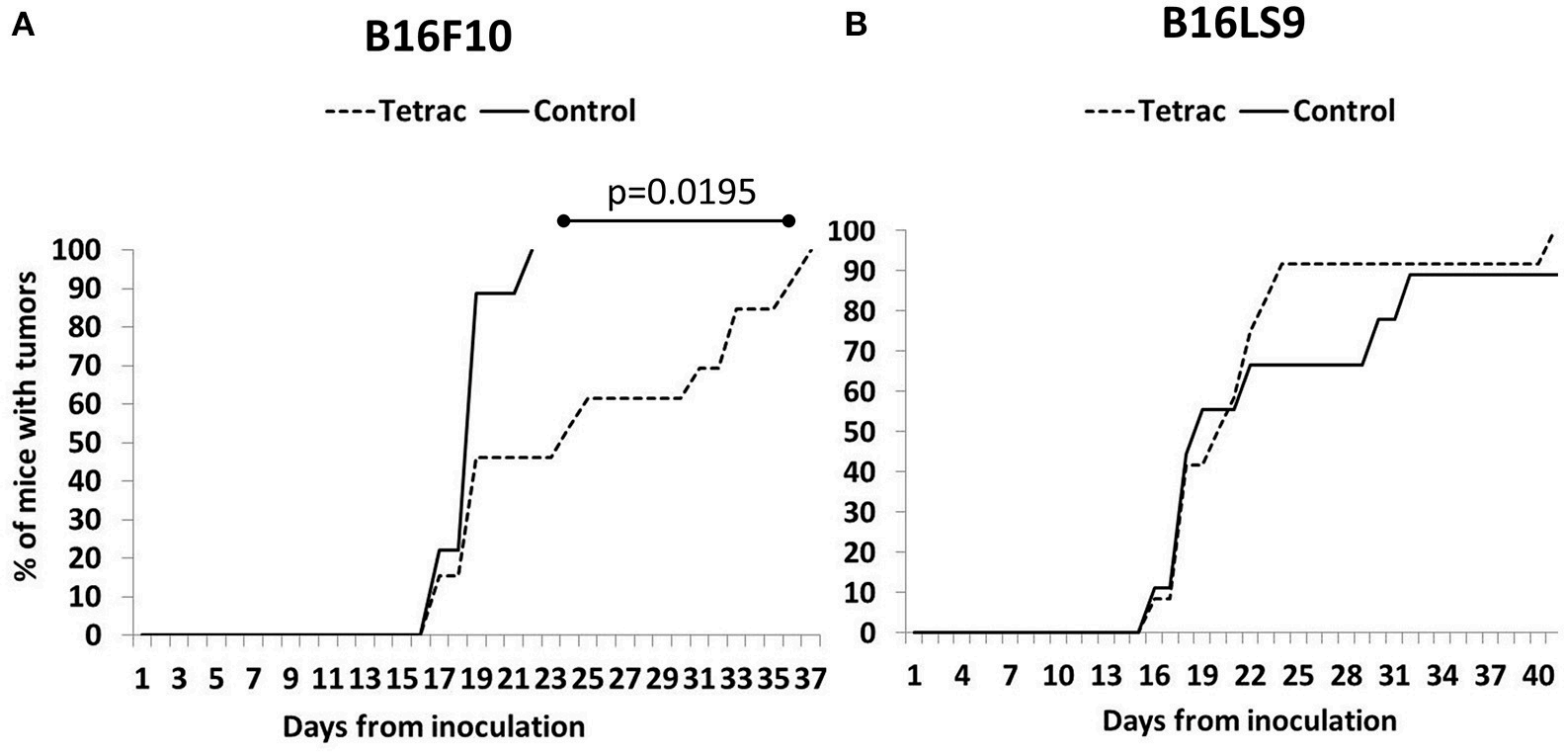
Tetrac delays the onset of ocular melanoma. Mice were inoculated with **(A)** integrin positive cells (B16F10 cells) but not in **(B)** integrin negative model (B16LS9 cells).

A small proportion of mice from both the B16F10 (Figure 5A) and B16LS9 group (Figure 5B) were diagnosed with intraocular tumors as early as 2 weeks from inoculation. However, investigating the control groups in both cell lines, tumors were evident in the B16F10 group, as a whole, in a significantly earlier and narrower time frame (15–21 days), as compared to the B16LS9 group (up to 40 days). Results further indicated a delay in tumor onset in the tetrac arm compared to the control group in the B16F10 mice model (Median 24 days to tumor onset versus median of 19 days, respectively). These results reached statistical significance by the logrank test (*p* = 0.0195). In the B16LS9 mice model, both the control and tetrac-treated groups exhibited a similar tumor onset rate.

**Figure 6:**
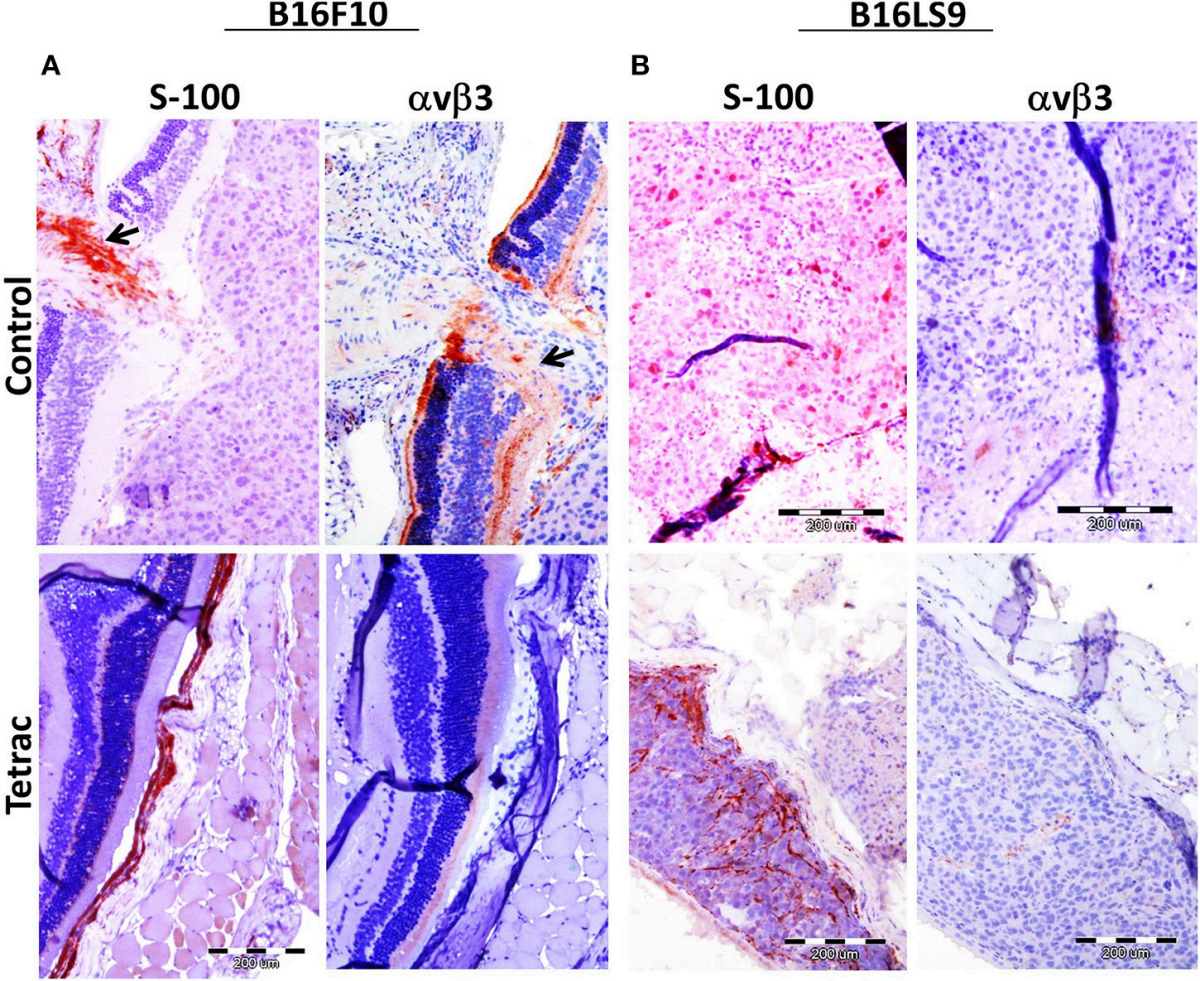
Enucleated murine eye of BALB/c White mice inoculated with **(A)** 100 B16F10 cells and **(B**) 100 B16LS9 cells in control and tetrac treated mice. Representative images of the intraocular tumor labeled for S100 (X10) and avb3 integrin (X10) are shown (positive (orange color) stained areas are indicated by arrows).

Mice were sacrificed after 90 days from study initiation, at which point eyes were enucleated and sent for pathological and immunohistochemical processing, including S100 (a melanoma marker) analysis and αvβ3 expression. Results indicate that in both the B16F10 (Figure 6A) and the B16LS9 (Figure 6B) cell models, the intraocular tumors were positive for S100 immunostaining, confirming the presence of melanoma cells. In accord with the flow-cytometry results, B16F10 (Figure 6A), but not B16LS9 (Figure 6B), were positively immunostained by the anti-integrin antibody. Lastly, in the B16F10 mice model, tetrac treatment clearly indicated a reduced level of S-100 and integrin staining, suggesting an inhibitory effect on tumor inoculation, growth and integrin expression.

## Discussion

The prognosis and survival of patients with ocular melanoma is poor, due to the metastatic nature of the disease and lack of effective therapeutic modalities. Reliable *in vivo* models that can reproduce and mirror human ocular melanoma are essential in order to better understand the disease's characteristics, particularly its metastatic behavior and susceptibility to potential treatment approaches. The significance of results from animal experiments relies on selection of an appropriate animal model. For ocular melanoma, several models exist, consisting of spontaneous, transgenic, and induced models. The latter, compared to the prior two, are easier to handle and are more reproducible (7). The most widely-used induced model in ocular melanoma research is the inoculation model, in which melanoma tumor cells are implanted into the animal's eye, with the mouse being the most widely used species. The small eye size limits the possibility of using routine eye examination. However, due to its cost-effectiveness, rapid reproduction rate, and the fact that 95% of the mouse genome is similar to that of humans, many experiments have been conducted that attempt to replicate in mice the tumor growth and migration behavior of human ocular melanoma. B16 mouse melanoma cell lines are commonly studied and have been successfully inoculated into syngeneic C57BL6 mice eyes (30, 31, 37). A subculture, the B16F10 cell line, which demonstrates high metastatic rate, has also been applied (32, 38–41). In all studies, about 10^5^ cells (range: 1–5 × 10^5^ cells) have been injected into the posterior or anterior chambers (31, 32, 34, 38, 42). When such cell numbers were used in our preliminary studies, eyes erupted at about 1 week after inoculation. In addition, tumor cells in these models are highly invasive and the tumor-bearing eye has to be enucleated at 7–14 days post inoculation; mice usually have to be sacrificed shortly after. By optimizing the number of cells required, we have established that an inoculation of 10^2^ cells is sufficient to develop an intraocular tumor and to exhibit an extended survival. It has been established that the B16F10 cell line metastasizes predominantly to the lungs (35) and this was confirmed in all of the animals sampled in our study, implying that it was the tumors' systemic spread that eventually killed the mice. In order to improve intraocular tumor visualization, we used Balb/C albino mice which were inoculated with 10^2^ B16F10 or B16LS9 melanoma cells. This latter cell line is derived from B16, is liver specific (43) and grows well in the eye (36). It has been previously reported that anterior chamber inoculation of B16LS9 cells in murine eyes results in iris melanoma, but is much less likely to metastasize to the liver than posterior compartment inoculation (44). We have observed that posterior inoculation of 10^2^ B16F10 or B16LS9 melanoma cells in the albino mice eye resulted in intraocular tumor behavior which did not metastasize and was associated with extended survival. Additionally, in the B16F10 model group, tumors developed at a significantly earlier stage compared to the time required for the B16LS9-inoculated mice.

The limited efficacy of current treatments in ocular melanoma, highlights the need for more novel treatment approaches (45). Accumulating data suggest that hyperthyroidism may increase the risk of certain non-ocular solid tumors, whereas hypothyroidism may delay disease onset and reduce aggressiveness of cancers (46, 47). We have recently observed a comparable association, both to tumor onset and survival, with states of thyroid function in the ocular melanoma B16F10 mouse model (24). We have further demonstrated in the same B16F10 melanoma cells as well as in an additional integrin positive human melanoma cell line (Malme-3M), the growth promoting effects of thyroid hormone *in vitro*. These results collectively are attributed to binding of the thyroid hormones to a receptor site on the plasma membrane integrin αvβ3 which may mediate the proliferative action of the hormones on tumor cells (9). The growth-promoting effects by the hormones encouraged us to study the potential of inhibitors of the thyroid hormone-integrin axis in the albino mouse ocular melanoma model. The approach of blocking this specific integrin is highly relevant to ocular melanoma as it is expressed in all tumor subtypes, including spindle, epithelioid, and mixed cell tumors (48). Of note, the B16F10 melanoma cells that were utilized in the present *in vivo* studies, highly express the αvβ3 integrin (24, 49), whereas B16LS9 cells were shown to possess low integrin expression. These findings are important additions for future study design and planning, especially when considering to target the integrin. We have shown that tetrac, a thyroid hormone derivative, clearly delayed tumor onset in mice receiving B16F10 (integrin positive) cells, compared to the untreated group, while in the B16LS9 cells (integrin negative) a similar rate of tumor onset was observed in both groups. Similar pharmacologic targeting of the hormone receptor with a liposomal modified formulation of tetrac was shown in the same B16F10 mice melanoma cells utilized by us (29) as well as in another human melanoma cell line (28). In both cell models tetrac was shown to bind to integrin αvβ3 resulting in reduction in cell proliferation and viability *in vitro* and as well as to reduce tumor growth and metastasis *in vivo*. Delay in tumor growth was observed with tetrac *in vitro* and *in vivo* in an array of tumor types [reviewed in (11)]. A number of laboratories have shown that specific inhibitors of αvβ3 slow growth of these melanoma, cells (50, 51). In contrast to these inhibitors, tetrac acts only at the remarkable thyroid hormone-tetrc receptor on αvβ3 to differentially regulate downstream the expression of a large number of genes related to the cell cycle, apoptosis and other cancer cell survival pathways (52–54). The plasma membrane thyroid hormone-tetrac receptor is exclusively located on integrin αvβ3 and no other alternate receptor was discovered (11). Tetrac binds to the αvβ3 integrin in two orientations and competitively displaces both 3,5,3′-triiodo-L-thyronine (T_3_) and T_4_, and thus inhibits their tumor-relevant activities (12, 55).

There are several limitations to this study. Although performed in the same manner for all of the experimental groups, the estimation of appearance of tumor after cell inoculation may be subject to error. However, we have used large enough cohorts in order to obtain significant differences between the experimental groups. Another limitation is the use of cutaneous-derived melanoma cells for ocular melanoma studies. However, intraocular inoculated B16 melanoma cells are commonly used to model ocular melanoma, including for evaluation of novel therapeutic approaches (31, 32, 34, 38, 42). For our specific integrin-targeted therapy, the blocking of the αvβ3 integrin, these cells were particularly appropriate, due to a positive high expression of this integrin.

To summarize, we have developed ocular melanoma mice models with an extended therapeutic window that may be exploited for preclinical evaluation of potential drugs. These models enabled us to assess a novel thyroid hormone-αvβ3-integrin targeted therapy, which delayed tumor onset. Tumor biopsies may serve for patient-based therapy following evaluation of the integrin abundance on the tumor cells. This, together with our published results which indicated a beneficial effect of a hypothyroid state on the primary ocular melanoma tumor (24), suggest that this mode of treatment may be administered as soon as the primary tumor is diagnosed.

## Author Contributions

IDF designed, preformed, analyzed, and interpreted the experimental data. OZ performed the experiments. OA-F designed, analyzed, and interpreted the experimental data. GT performed the ultrasound and doppler measurements. DS performed the statistical analysis. IDF, IF, AH, PD, ME and OA-F wrote the manuscript. All authors read and approved the final manuscript.

### Conflict of Interest Statement

PD is stockholder and officer in a company developing modified forms of tetrac as anticancer agents.

The remaining authors declare that the research was conducted in the absence of any commercial or financial relationships that could be construed as a potential conflict of interest.
