# Supplementary figures and images for: Tetrac Delayed the Onset of Ocular Melanoma in an Orthotopic Mouse Model
Source: Front Endocrinol (Lausanne). 2019 Jan 8;9:775. doi: 10.3389/fendo.2018.00775 (PMC6331424; doi:10.3389/fendo.2018.00775)

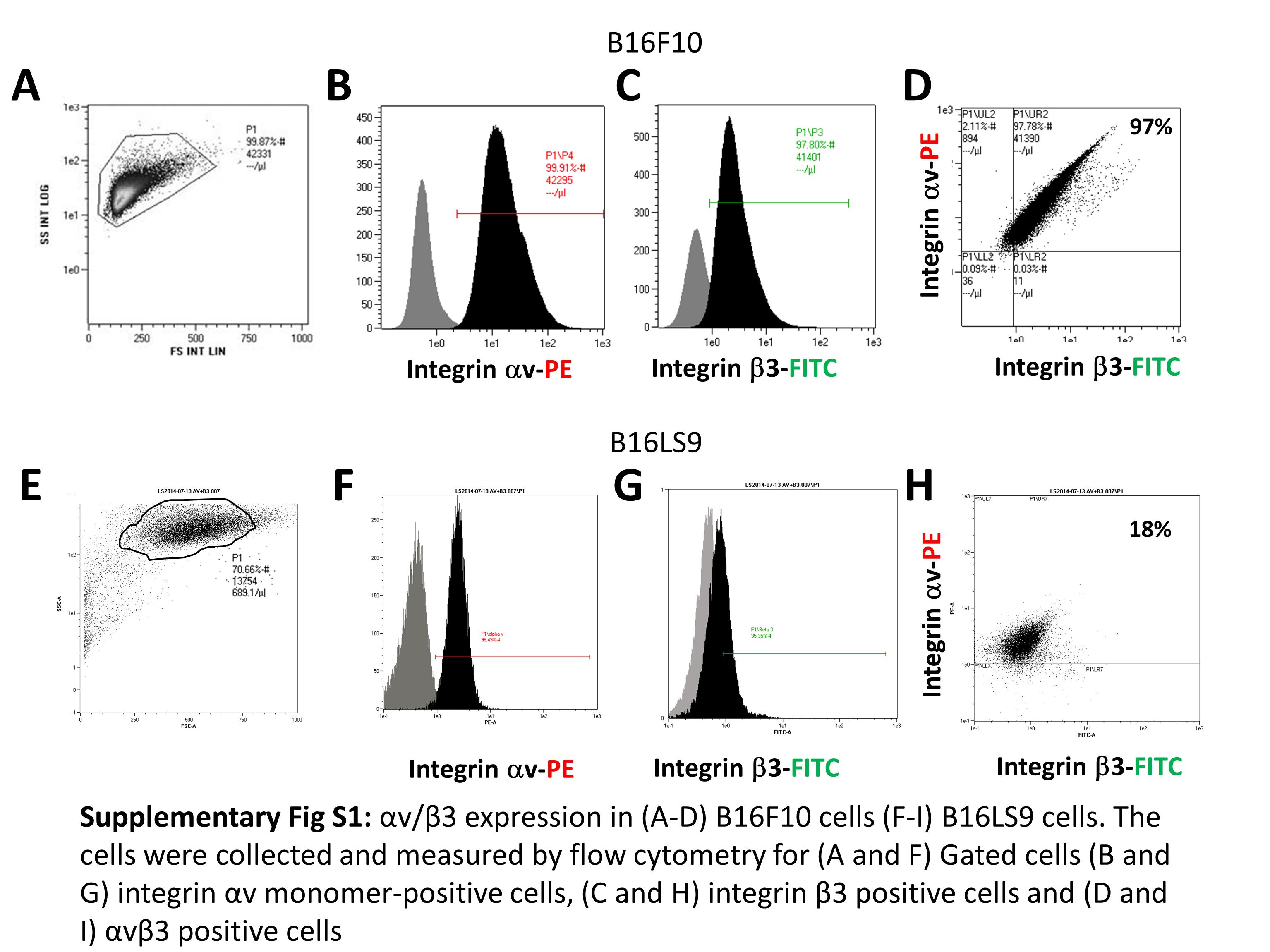

Supplement: Supplementary file 1 [file Image_1.tif]
